# External validation and biomarker assessment of a high-risk, data-driven pediatric sepsis phenotype characterized by persistent hypoxemia, encephalopathy, and shock

**DOI:** 10.21203/rs.3.rs-3216613/v1

**Published:** 2023-08-02

**Authors:** Mihir R. Atreya, Tellen D. Bennett, Alon Geva, E. Vincent S. Faustino, Colin M. Rogerson, Riad Lutfi, Natalie Z. Cvijanovich, Michael T. Bigham, Jeffrey Nowak, Adam J. Schwarz, Torrey Baines, Bereketeab Haileselassie, Neal J. Thomas, Yuan Luo, L. Nelson Sanchez-Pinto, Novel Data-Driven Sepsis Phenotypes in Children Study and the Genomics of Pediatric Septic Shock Investigators.

**Affiliations:** 1 Division of Critical Care Medicine, Cincinnati Children’s Hospital Medical Center and Cincinnati Children’s Research Foundation, Cincinnati, 45229, OH, USA.; 2 Department of Pediatrics, University of Cincinnati College of Medicine, Cincinnati, OH 45267, USA.; 3 Departments of Pediatrics and Biomedical Informatics, University of Colorado School of Medicine, Aurora, CO; 4 Department of Anesthesiology, Critical Care, and Pain Medicine, Boston Children’s Hospital, Boston, MA; Computational Health Informatics Program, Boston Children’s Hospital, Boston, MA; 5 Department of Pediatrics, Yale School of Medicine, New Haven, CT; 6 Department of Pediatrics, Riley Hospital for Children, Indianapolis, IN 46202, USA.; 7 Department of Pediatrics, UCSF Benioff Children’s Hospital Oakland, Oakland, CA 94609, USA; 8 Department of Pediatrics, Akron Children’s Hospital, Akron, OH 44308, USA.; 9 Department of Pediatrics, Children’s Hospital and Clinics of Minnesota, Minneapolis, MN 55404, USA.; 10 Children’s Hospital of Orange County, Orange, CA 92868, USA.; 11 University of Florida Health Shands Children’s Hospital, Gainesville, FL 32610, USA.; 12 Department of Pediatrics, Lucile Packard Children’s Hospital Stanford, Palo Alto, CA 94304, USA.; 13 Department of Pediatrics, Penn State Hershey Children’s Hospital, Hershey, PA 17033, USA.; 14 Department of Pediatrics, Northwestern University Feinberg School of Medicine, Chicago, 60611, IL, USA.; 15 Department of Health and Biomedical Informatics, Northwestern University Feinberg School of Medicine, Chicago, 60611, IL, USA.

**Keywords:** Critical Care, Pediatrics, Sepsis, Multiple Organ Dysfunction Syndrome, Precision Medicine, Biomarkers, Systemic Inflammation, Endothelial Dysfunction

## Abstract

**Objective:**

Identification of children with sepsis-associated multiple organ dysfunction syndrome (MODS) at risk for poor outcomes remains a challenge. Data-driven phenotyping approaches that leverage electronic health record (EHR) data hold promise given the widespread availability of EHRs. We sought to externally validate the data-driven ‘persistent hypoxemia, encephalopathy, and shock’ (PHES) phenotype and determine its association with inflammatory and endothelial biomarkers, as well as biomarker-based pediatric risk-strata.

**Design:**

We trained and validated a random forest classifier using organ dysfunction subscores in the EHR dataset used to derive the PHES phenotype. We used the classifier to assign phenotype membership in a test set consisting of prospectively enrolled pediatric septic shock patients. We compared biomarker profiles of those with and without the PHES phenotype and determined the association with established biomarker-based mortality and MODS risk-strata.

**Setting:**

25 pediatric intensive care units (PICU) across the U.S.

**Patients:**

EHR data from 15,246 critically ill patients sepsis-associated MODS and 1,270 pediatric septic shock patients in the test cohort of whom 615 had biomarker data.

**Interventions:**

None

**Measurements and Main Results:**

The area under the receiver operator characteristic curve (AUROC) of the new classifier to predict PHES phenotype membership was 0.91(95%CI, 0.90–0.92) in the EHR validation set. In the test set, patients with the PHES phenotype were independently associated with both increased odds of complicated course (adjusted odds ratio [aOR] of 4.1, 95%CI: 3.2–5.4) and 28-day mortality (aOR of 4.8, 95%CI: 3.11–7.25) after controlling for age, severity of illness, and immuno-compromised status. Patients belonging to the PHES phenotype were characterized by greater degree of systemic inflammation and endothelial activation, and overlapped with high risk-strata based on PERSEVERE biomarkers predictive of death and persistent MODS.

**Conclusions:**

The PHES trajectory-based phenotype is reproducible, independently associated with poor clinical outcomes, and overlap with higher risk-strata based on validated biomarker approaches.

## INTRODUCTION:

Sepsis-associated multiple organ dysfunction syndrome (MODS) is a major cause of morbidity among children across the globe (1). Identification of high-risk patients may lead to the deployment of targeted strategies, beyond antibiotics and organ support, which may improve clinical outcomes. In the previous decade, serum biomarker based risk-models predictive of pediatric sepsis mortality (PERSEVERE) have been prospectively validated (2, 3). Recent iterations which incorporate endothelial biomarkers (PERSEVERENCE) have shown promise in identifying those at risk of persistent MODS (4). Yet, real-time risk-stratification is a challenge as we currently lack point-of-care assays for biomarker measurement. Moreover, the infrastructure required to support such an approach may not become ubiquitously available, especially in non-quaternary healthcare settings. In contrast, electronic health record (EHR) data are readily available and may serve as a useful surrogate to identifying high-risk patients.

We recently derived and validated a data-driven phenotype of high-risk sepsis-associated MODS based on organ dysfunction trajectories using EHR data (5). This phenotype, which we called the ‘persistent hypoxemia, encephalopathy, and shock’ (PHES) phenotype based on its clinical characteristics, was independently associated with worse clinical outcomes and differential response to common adjuvant therapies. In the current study, we sought to externally validate the prognostic utility of our phenotyping approach, test association between biomarkers of systemic inflammation and endothelial activation and PHES phenotype membership, and assess the overlap with established biomarker based risk-strata in a large prospective observational cohort of pediatric septic shock.

## METHODS:

Reporting of observational cohort studies was performed using the Strengthening the Reporting of Observational Studies in Epidemiology (STROBE) reporting guideline.

### Derivation and validation set:

We used data from a retrospective, multicenter, observational cohort study (*Novel Data-Driven Sepsis Phenotypes in Children*) (5). The institutional review board (IRB) at Ann & Robert H. Lurie Children’s Hospital of Chicago served as the central IRB for this study (IRB# 2019–2481, approved on 2/13/2019 with a waiver of consent). Briefly, children 0 to 18 years old admitted to one of 13 participating U.S. pediatric intensive care units (PICUs) between January 1, 2012 and January 1, 2018 were included. Data for patients who had a confirmed or suspected infection were extracted from the EHRs of the participating institutions, of whom 15,246 patients had sepsis-associated MODS and included in the current study.

### Test set:

We used data from 1,270 patients enrolled in a prospective observational cohort of pediatric septic shock patients (*Genomics of Pediatric Septic Shock Cohort*) which has been described previously (3, 6). The study protocol was approved by IRB of participating institutions (Cincinnati Children’s Hospital IRB ID: 2008–0558, Initial Approval 5/9/2002). All research involving human participants were in accordance with the ethical standards of the IRBs and with the 1964 Helsinki declaration and its later amendments. Briefly, patients under the age of 18 years were recruited from 16 PICUs across the U.S. between 2003 and 2023.

### Phenotype assignment:

**Figure 1.**
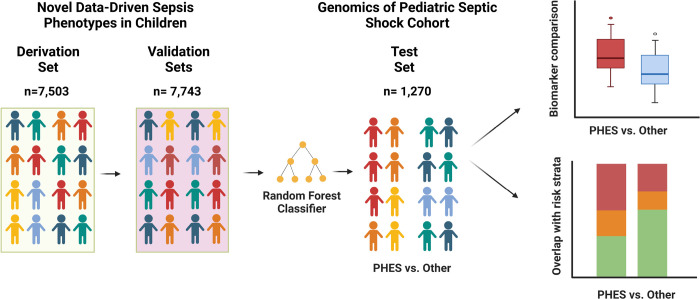
Overview of study detailing study subjects in derivation, validation, and test cohorts.

The PHES phenotype designation was based on the trajectory of the six pediatric Sequential Organ Failure Assessment subscores (pSOFA) during the first 72 hours after PICU admission (5). However, the test cohort did not collect all variables used to assign pSOFA subscores. Specifically, SpO_2_/FiO_2_ levels were not collected, PaO_2_/FiO_2_ levels were dichotomized as ≥ or < 250, Glasgow coma scale scores dichotomized as ≥ or < 8, and there were no bilirubin levels. Thus, we split patients in EHR dataset (n=7,503) to derive a random forest classifier using the pSOFA cardiovascular, renal, and coagulation sub-scores, and a modified respiratory subscore based on the dichotomized PaO_2_/FiO_2_ levels, and validate its performance at predicting PHES phenotype membership (n=7,743). We then used the classifier to assign phenotypes in the test set and compared clinical characteristics and outcomes among those with and without the phenotype of interest. The overall approach is summarized in Figure 1. The primary outcome of interest was complicated course, a composite that included patients who had died by or had persistence of ≥ 2 organ dysfunctions on day 7 of septic shock (4). The secondary outcome was 28-day mortality. Multivariate models adjusting for age, severity of illness based on the Pediatric Risk of Mortality (PRISM) III score, and immunocompromised status were used to test the independent association between phenotype and outcomes. All biomarkers were previously measured and methods for risk-stratification are detailed in the **Online Supplement.**

### Statistical analyses:

We used R version 4.0 and Minitab Software (PA, USA, version 21.1.0) for data analyses, and GraphPad Prism (CA, USA, version 9) to generate figures. One-way analysis of variance (ANOVA) was used to compare biomarkers between phenotypes. Multivariate models were used to assess independent association between biomarkers and phenotype after adjusting for confounders and performing backward selection at an alpha threshold of <0.05. Association of phenotypes with establish mortality and MODS risk-strata were performed using χ^2^ test.

## RESULTS:

The random forest classifier had an area under the curve of 0.91 (95%CI: 0.90–0.92) at predicting PHES phenotype in the validation set. A total of 513 out of 1,270 (40.3%) patients were assigned to the PHES phenotype in the test set. Comparison of demographics, clinical characteristics, and outcomes between those with and without PHES phenotype across cohorts are shown in **Table 1**. PHES phenotype membership was independently associated with higher odds of complicated course (adjusted odds ratio [aOR] of 4.1, 95%CI: 3.1–5.4) and 28-day mortality (aOR of 4.8, 95%CI: 3.1–7.2) after adjusting for confounders in the test set, detailed in **Online Supplement, Table 1.**

Of the 1,270 patients in the test set, 615 (48%) patients had complete biomarker data available. Inflammatory markers IL-8, HSP70, CCL3, CCL4, GZMB and IL-1a were higher among patients with the PHES phenotype vs. other septic shock patients (p<0.05, **Supplemental Digital Content, Figure 1).** The endothelial markers sTM, Angpt-2, Angpt-2/Angpt-1, Angpt-2/Tie-2, and ICAM-1 were also higher among patients with PHES phenotype vs. other septic shock patients (p<0.05, **Supplemental Digital Content, Figure 2**). There were no statistically significant differences between groups when comparing MMP-8, Angpt-1, Tie-2, VCAM-1, and PECAM-1 concentrations. After adjusting for confounders, Angpt-2/Angpt-1 ratio, HSP70, Angpt-1, and ICAM-1 concentrations were independently associated with PHES phenotype (**Online Supplement, Table 2**). **Supplemental Digital Content, Figure 3** shows the association between the PHES phenotype and PERSEVERE-II mortality- and PERSEVERENCE MODS- risk-strata, respectively. A greater proportion of those classified as PHES phenotype were also categorized as high-risk (20% vs. 9%) and intermediate risk (25% vs. 18%) based on PERSEVERE-II mortality risk (p <0.001) and high-risk (37 % vs. 21 %) based on PERSEVERENCE persistent MODS risk (p <0.001). However, nearly 50% and 25% of patients with the PHES phenotype were categorized as low-risk per biomarker-based mortality- and MODS-risk strata respectively.

## DISCUSSION

In this study, we externally validated the data-driven PHES phenotype in a large prospective cohort of pediatric septic shock patients. We show that the PHES phenotype was associated with a biomarker profile reflective of greater systemic inflammation and endothelial activation — a pattern common among data-driven phenotypes of children and adults with sepsis and acute respiratory distress syndrome (ARDS) (7, 8). Correspondingly, the PHES phenotype showed some overlap with patients designated as high-risk based on established biomarker-based approaches. It is worth noting that traditional criteria such as “septic shock” appear to have low specificity for identifying such high-risk subgroup of patients. Future ‘omic’ studies that evaluate whether there are differences between phenotypes identified or overlap with established endotypes of critical illness may serve to identify latent endo-phenotypes within the broader phenotypic designations with potential therapeutic implications.

Several recent studies have used trajectory-based approaches that leverage EHR data to characterize high-risk phenotypes of sepsis (9, 10). To the best of our knowledge our study is the first to directly compare outputs of trajectory-based phenotypes with biomarker-based risk-stratification approaches. The main strength of our study is the large datasets used. The limitations of our study are (1) the test cohort did not collect all the variables included in the pSOFA subscores and this required a modified approach to assign phenotype membership. Nevertheless, our classifier using the modified pSOFA subscores demonstrated robust discrimination at predicting PHES phenotype and the patients classified as PHES phenotype in the test cohort had very similar characteristics when compared to the derivation and validation sets; (2) biomarkers analyzed was limited to those selected for study in the test set; (3) data-driven phenotypes and biomarker based risk-stratification approaches were non-synonymous. Although expected, our data suggest that while a useful surrogate, data-driven phenotyping approaches in isolation may lack the specificity of biomarkers and thus inherently limited in their predictive capabilities.

## CONCLUSION:

The persistent hypoxemia, encephalopathy, and shock (PHES) phenotype of pediatric sepsis is reproducible and independently associated with increased risk of mortality and persistent MODS. This phenotype had a biomarker profile characterized by systemic inflammation and endothelial activation, and demonstrates some overlap with patients deemed high-risk using validated biomarker-based risk-stratification. Future research is necessary to determine whether real-time risk stratification using EHR data can be used to accurately identify at-risk patients for prognostic enrichment in clinical trials and targeted management.

## Key points:

### Question:

We sought to externally validate an organ dysfunction-based pediatric sepsis phenotype in a prospective cohort of pediatric septic shock patients, determine its association with biomarkers of systemic inflammation and endothelial activation, and compare the phenotype with established biomarker-based risk-strata.

### Findings:

The persistent hypoxemia, encephalopathy, and shock (PHES) phenotype was reproducible and independently associated with risk of poor clinical outcomes in the prospective cohort, had a biomarker profile consistent with systemic inflammation and endothelial activation, and had overlap high-risk strata of biomarker-based models.

### Meaning:

The PHES sepsis phenotype is reproducible in an external cohort and can help identify children at high-risk of poor outcomes.
